# Evaluating the potential of *Hibiscus sabdariffa* beverage to address the prevalence of iron deficiency in sub-Saharan Africa

**DOI:** 10.1016/j.lwt.2023.115433

**Published:** 2023-10-01

**Authors:** Ade O. Oyewole, Levente L. Diosady

**Affiliations:** Department of Chemical Engineering and Applied Chemistry, University of Toronto, 200 College Street, Toronto, ON M5S 3E5, Canada

**Keywords:** *Hibiscus sabdariffa* L., Iron deficiency, Iron-bioaccessibility, Iron-polyphenol complex

## Abstract

The potential of *Hibiscus sabdariffa* L. beverage as a dietary iron source for sub-Saharan Africans was investigated. The target was to provide 6 mg of iron through 250 mL of the beverage daily. However, the iron content of the dried hibiscus calyces was determined to be 9.73 ± 0.31 mg/100 g and from that only ∼30% was extractable, resulting in 0.93 ± 0.19 mg Fe/250 mL of the selected beverage formulation. Therefore, ferrous sulfate was explored as a fortificant. The beverage contains polyphenols which could form non-absorbable chelation complexes with iron during digestion. Subsequently, the effect of polyphenols on the bioaccessibility of native and added iron was assessed using spectrophotometric methods. The presence of iron-polyphenol complexes in samples of the unfortified and fortified beverages, adjusted to pH 6.5 (pH at site of iron absorption in the gut) was established. However, only ∼25% of the added iron was found to be bound in the complex. It was shown that the viability of *H. sabdariffa* L. beverage as an iron source is impacted by extraction losses and the inhibitory effect of polyphenols. Nonetheless, if iron-polyphenol complexation was reduced/prevented then, a fortified hibiscus beverage could be a useful iron source.

## Highlights

• Fortified *H. sabdariffa* beverage could potentially improve iron intake and uptake.
• ∼70% of unextracted iron renders intake from unfortified beverage insufficient.
• ∼75% of added iron remained unbound and thus was available for absorption.
• Inhibitory effect of beverage's polyphenols on native/added iron was estimated.
• Describes a viability-screening approach for iron-fortified polyphenol-rich “foods".

## Graphical abstract

**Figure:**
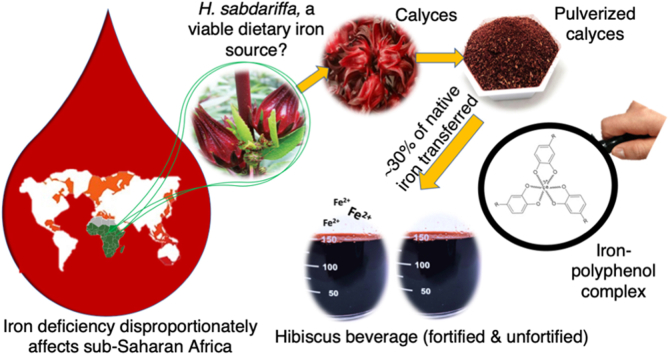

## 1 Introduction

*Hibiscus sabdariffa* L*.* is cultivated and consumed in many countries in the tropical and sub-tropical regions of the world including sub-Saharan Africa, South Asia, East Asia, the Caribbean, and parts of North America (Abou-Arab et al., 2011; Majdoub et al., 2021; Nguyen & Chuyen, 2020, p. 2). *H. sabdariffa* L. belongs to the Malvaceae family and its common names include hibiscus, red sorrel or roselle*,* kakarde and bissap (Majdoub et al., 2021; P. Singh et al., 2017).

Recently, the plant has gathered interest because of its economic potential (Abou-Arab et al., 2011; Islam et al., 2016) as well as reports of its nutritional (P. Singh et al., 2017) and therapeutic benefits (Majdoub et al., 2021). Some of the reported therapeutic applications of the plant include the management of cancer, diabetes, high blood pressure and cardiovascular diseases (Islam et al., 2016; Sáyago-Ayerdi et al., 2007; P. Singh et al., 2017). Additionally, other authors have reported on the nutritional composition of *H. sabdariffa* L. Although this varies with the site of cultivation, generally, the plant is regarded as a valuable source of vitamins and minerals, including iron, calcium, phosphorous, ascorbic acid and carotene, as well as other bioactive compounds (Islam et al., 2016; Sáyago-Ayerdi et al., 2007).

The calyces are reportedly the most valuable part of the plant, useful as the raw materials for several products such as food colorants, jellies, juices, and jam (Abou-Arab et al., 2011; Salami & Afolayan, 2020). The bright red aqueous extract of the plant's calyces is quite popular as a hot or cold beverage, and it is considered a staple in many homes in the sub-Saharan Africa region (Abou-Arab et al., 2011; Islam et al., 2016; Salami & Afolayan, 2020; Sáyago-Ayerdi et al., 2007). The beverage is also commercially marketed based on its refreshing taste, cost, and perceived healthy, natural ingredients (Islam et al., 2016; Salami & Afolayan, 2020). Common names for commercial hibiscus (*Hibiscus sabdariffa* L*.*) beverages include “Zobo” in Nigeria (Salami & Afolayan, 2020) and “Flor de Jamaica” in Mexico (Sáyago-Ayerdi et al., 2007).

Gardner and Kassebaum (2020) reported that anemia adversely affected 22.8% of the world's population in 2019. It has also been established that dietary iron deficiency (ID) is a leading contributor to the burden of anemia, accounting for over 50% of anemia cases worldwide (Black, 2003; Pasricha et al., 2013). Iron deficiency primarily affects low- and middle-income countries (LMIC), undermining the health and productivity of at-risk groups such as premenopausal women and preschool-age children (Yang et al., 2018). The devastating consequences of ID include high infant and maternal mortality rates, cognitive impairment, low birth weight, impaired growth and development in children and pregnant women, and reduced physical capacity and work productivity which inevitably adversely impacts the subsistence and economic situation of the affected populations (Darnton-Hill et al., 2017; Pasricha et al., 2021; Yang et al., 2018).

Despite concerted scientific and economic efforts, in the last few decades, there has yet to be a significant decrease in the prevalence of ID in LMICs (Stevens et al., 2013, 2022; World Health Organization, 2008). In a 2020 review, the World Health Organization (WHO) recommended that different strategies must be combined to meet the global nutrition target of the World Health Assembly: a 50% reduction in the prevalence of ID among vulnerable groups by 2025 (World Health Organization, 2020).

Sub-Saharan Africa has a staggering number of rural-poor households; therefore, affordability, accessibility, and availability limit the effectiveness of strategies such as supplementation and food diversification to address ID (Mwangi et al., 2021). Conversely, fortification of staple foods is considered a low-cost approach to curb nutritional deficiencies like ID among large populations and it has been demonstrated to be effective for LMICs (World Health Organization, 2020). Vulnerable populations in sub-Saharan Africa are largely dependent on plant-based diets, therefore their diets consist mostly of non-heme iron (Mwangi et al., 2021). This less bioaccessible form of iron is known to interact easily with iron absorption inhibitors present in plant food sources: phytates and polyphenols (McGee & Diosady, 2018a). Therefore, improving the bioaccessibility of non-heme iron in plant-based diets should be part of the approach to reduce the prevalence of ID in the region. The inhibitory effect of phytates on the absorption of iron as well as approaches to circumvent this have been well reported on, however, there are few reports on preventing iron-polyphenol interactions.

Polyphenols, specifically, those bearing either the galloyl or catechol groups (ortho-dihydroxy phenolic compounds) are known to inhibit iron absorption by forming chelation complexes (Hurrell & Egli, 2010; Raes et al., 2014). These complexes cannot be digested by humans, the trapped iron therefore becomes non-bioaccessible (van der Merwe et al., 2019). To develop strategies to prevent the formation of these complexes there is the need to assess the effect of polyphenols on the iron content of the “food” vehicle of interest. This is especially important when considering a complex biological material as the delivery vehicle, more so one that reportedly contains iron absorption enhancers, such as ascorbic acid, that could counter the inhibitory effect of polyphenols.

Several studies have investigated the potential of products from *H. sabdariffa* L. to address widespread ID in sub-Saharan Africa (Kubuga et al., 2019; Peter et al., 2017). Some have even reported the application of the calyces of the plant as a food-to-food fortificant to improve both the intake and uptake of iron (van der Merwe et al., 2019). A few have reported the anti-anemic properties of hibiscus beverage for the local management of ID (El-Hashash, 2021; Falade et al., 2005). However, no known studies have attempted to validate the hibiscus beverage's potential to address iron deficiency at a population level by evaluating iron intake and uptake from the beverage.

Consequently, the overarching aim of this study is to develop an approach to assess the potential of delivering 6 mg, 30% of 18 mg (recommended dietary allowance (RDA) of iron for premenopausal women), through 250 mL of the beverage daily, in a bioaccessible form. This would be accomplished by:

• Evaluating how much iron is extracted from the raw hibiscus calyces into the beverage.
• Adding an iron salt (fortificant) to make up the targeted quantity of iron (6 mg) via 250 mL of the beverage.
• Using spectrophotometry, assess the possible effect of iron-polyphenol interactions on the bioaccessibility of the native and added iron at pH 6.5: the pH value at the site of iron absorption in the gastrointestinal (GI) tract.

For this study, the daily serving of the hibiscus beverage was assumed to be 250 mL because the global average intake per person for sugar-sweetened beverages, reported in 2010, was 8 oz or 236.5 mL/day (G. M. Singh et al., 2019).

## 2 Materials and experimental methods

### 2.1 Materials

Air-dried and ground *Hibiscus sabdariffa* L*.* calyces were sourced from and authenticated by the National Horticultural Research Institute (NIHORT) Ibadan (Oyo State, Nigeria).

Folin and Ciocalteu's phenol reagent, gallic acid, iron standard for ICP OES and ferrous sulfate heptahydrate, were purchased from Sigma-Aldrich Canada Co. (Oakville ON, Canada). Sodium carbonate was purchased from Fisher Scientific (Toronto ON, Canada). Ferric chloride hexahydrate was purchased from VWR International LLC. (Mississauga ON, Canada). Sodium hydroxide and hydrochloric acid were purchased from Caledon Laboratories (Caledon ON, Canada) and BioShop Canada Inc. (Burlington ON, Canada) respectively. For the experiments, all reagents were analytical grade, unless otherwise stated.

### 2.2 Experimental methods

#### 2.2.1 Iron analysis

The total iron content of the dry hibiscus plant calyces, as well as the hibiscus beverage samples, were determined using the method described in the Elemental Analysis Manual by the U.S. Food and Drug Administration agency (Mindak & Dolan, 2010) with slight modifications. Briefly, 0.5 g of samples were placed in microwave digestion vessels, and 10 mL of conc. Nitric acid (67–70%) was added to each vessel and sealed. Samples were digested in a microwave digestion system (CEM MARS 6, John Morris Scientific Pvt., Ltd.) at a raised temperature of 200–210 °C. The resulting solutions were then transferred into 25 mL flasks and brought to volume with deionized water. Samples were further diluted to a known volume (1.5:10) with 5% w/v nitric acid based on the estimated iron content. An Inductively Coupled Plasma Optical Emission Spectrophotometer (iCAP Pro ICP OES, Thermo Scientific) was used to determine iron contents, with 1000 mg/L iron standard (Merck) solution used for calibration.

#### 2.2.2 Polyphenolic content determination

The polyphenolic content of the aqueous extract of *H. sabdariffa* L*.* calyces was determined by the Folin-Ciocalteu method as reported by Singleton and Rossi (1965). First, to generate a calibration curve, Folin and Ciocalteu's reagent was added to six gallic acid solutions of different concentrations within the 0.001–0.01 g/L range, mixed thoroughly and left standing for 5 min. Saturated sodium carbonate solution was then added into each mixture, again thoroughly mixed, and thereafter RO water was added to obtain the desired final concentrations. Samples were kept at room temperature; after 30 min, the absorbance of each sample against a blank containing no gallic acid was measured at 765 nm using a UV-VIS spectrophotometer (Varian Cary 50). The total phenolic contents were reported as gallic acid equivalents in mg GAE/mL or g GAE/L.

#### 2.2.3 Hibiscus beverage extraction process

As there is no standardized recipe for preparing the hibiscus beverage, calyces weight to final beverage volume (w/v%), within the range found in literature, were compared. Specifically, to make 100 mL of the extract, 3.125, 6.250, or 12.500 g of the pulverized hibiscus calyces were weighed and dispersed in 80 mL of reverse osmosis (RO) water. The mixture was brought to a boil in about 30 min. The suspension was cooled for 30 min, and then the solids were removed using a 3-inch mini-strainer. After this, the collected solution was vacuum filtered using a porcelain Buchner funnel with 11 cm Fisher brand P8 qualitative filter paper (particle retention: 20–25 μm). Filtrates were made up to 100 mL with RO water in a volumetric flask. The beverage samples were stored at ∼4 °C pending analysis.

#### 2.2.4 Determination of the extractability of iron

The three selected weights of the hibiscus calyces, 3.125 g, 6.250 g, and 12.500 g were treated as discussed above to make beverages differing in concentration. However, the solid residues from the straining and vacuum filtration steps were combined for this set of experiments. Then, to maximize the extraction of soluble iron, the combined residues were washed thrice with 100 mL of RO water and re-filtered each time. Next, the collected filtrates were also combined, made up to 500 mL, and divided between 2 pre-weighed 300 mL capacity ceramic crucibles. Filtrates were oven-dried for 24 h at 105 °C. After drying, crucibles were re-weighed to determine the weight of the dried samples, which were then transferred to airtight scintillation vials pending iron content determination using the ICP OES.

The extractability of the iron (Fe), defined as the percentage of iron transferred from the calyces to the beverage during the extraction process was determined using the equation below:

$$\%Fe\;Extracted=\frac{Extracted\;Fe}{Total\;Fe\;Content}\times 100$$

Where, Extracted Fe = wt. of dried filtrate (g) × Filtrate Fe content (mg/g).

Total Fe Content = wt. of sample (g) × Hibiscus calyces Fe content(mg/g).

And, Filtrate Fe Content (mg/g) and Hibiscus calyces Fe content (mg/g) were the iron contents determined by analyzing the iron concentration in the filtrate sample and dried and pulverized calyces respectively, using the ICP OES.

All analyses were done in triplicates.

#### 2.2.5 Effect of extraction conditions on iron and polyphenol yield

Table 1 presents a 2^3^ full factorial experiment designed to determine the conditions that would result in the highest iron extraction without deviating too far from the processes used in the commercial preparation as reported by Salami and Afolayan (2020). Apart from the effect of the concentration of the solid calyces relative to the final beverage volume (w/v%), this experimental design also considered the effect of temperature and steep time on the iron yield after extraction. The polyphenolic yield was also monitored concurrently.

**Table 1:** A 2^3^ Design of Experiment (DOE) showing the lower and upper levels of each factor considered.

Run^a^	Weight/Volume %	Temperature (°C)	Steep Time (h)
A_L_	6.25	90	24.0
B_L_	6.25	90	0.5
C_L_	6.25	RT^b^	24.0
D_L_	6.25	RT^b^	0.5
A_U_	12.50	90	24.0
B_U_	12.50	90	0.5
C_U_	12.50	RT^b^	24.0
D_U_	12.50	RT^b^	0.5

DOE to determine conditions that would result in the most iron yield, the polyphenolic content was also monitored concurrently.

a L: lower concentration level; U: upper concentration level.

b RT – Room temperature (26.1–28 °C).

The beverage samples which gave the highest iron yield from the factorial experiment for both concentration levels were identified and were further screened to select a “working beverage sample.”

#### 2.2.6 Selection of extraction conditions

The iron and polyphenolic content of two commercially marketed hibiscus beverage samples sourced from Nigeria, West Africa, labeled “Zobo”–banana and “Zobo”–coconut, were determined. These values were then compared to those of the samples at each concentration level of the factorial experiment, identified as having the highest iron yields. The “working beverage sample” was selected based on iron yield and polyphenolic content closest to those of the commercially marketed hibiscus beverages.

#### 2.2.7 Iron fortification of the hibiscus beverage

The selected extract was fortified by direct addition of ferrous sulfate heptahydrate (FeSO_4_.7H_2_O), chosen as the fortificant because of its high-water solubility and bioavailability (Hurrell & Egli, 2010). An aliquot of freshly prepared 0.0125 M stock solution of ferrous sulfate was added to a volumetric flask three-quarters full of the extract. It was vigorously shaken, then topped with the remaining hibiscus beverage. The target was a final iron concentration of ∼0.43 mM in the fortified hibiscus beverage. The fortified beverage was stored at ∼4 °C pending analysis.

#### 2.2.8 Spectrophotometric quantitation of iron-polyphenol complexes

The colorimetric assay of phenolic compounds, developed by McGee and Diosady (2018b) based on the ferric chloride test (Pavia et al., 2017), was simplified and adapted for this study. The iron-polyphenol chelation complexes formed in the unfortified and fortified hibiscus beverages were quantified using a microplate reader (Tecan Infinite 200 pro) within the 400–800 nm range. Typically, the peak for the colored ligands formed when iron cations and polyphenols interact occurs between 542 and 561 nm for gallates; 561 and586 nm for catecholates (McGee & Diosady, 2018a; Perron et al., 2010).

The interaction between gallic acid and ferrous sulfate served as the model reaction and was used to prepare standards. Calibration curves were generated by keeping the concentration of the gallic acid solution at the estimated concentration of polyphenols in the “working beverage sample” while varying the concentration of the iron salt. For the unfortified beverage, the selected range was 0.01 mM–0.05 mM of ferrous sulfate in steps of 0.01 mM, while the range was 0.1 M–0.5 M in steps of 0.1 M for the fortified beverage. The pH values of standards and samples were adjusted to 6.5 ± 0.2 before analysis to mimic the pH of the small intestine: the site of iron absorption (Tissot et al., 2014). A standard with no iron was used to zero the absorbance scans of the other standards. For the beverage samples, reverse osmosis water, adjusted to the same pH as the samples served as the blank. Net absorbances were plotted against the iron salt concentration, and the estimated quantity of the iron-polyphenol complexes formed were expressed in terms of ferrous sulfate in gallic acid equivalents (mM FeSO_4_/GAE).

## 3 Results and discussion

### 3.1 Iron extractability

The amount of iron that can be extracted using the conventional method of producing the hibiscus beverage was estimated to determine whether the hibiscus beverage, without iron fortification, could contribute significantly to the iron uptake of consumers. Using the ICP OES, the iron content of the pulverized hibiscus calyces was determined to be 9.7 ± 0.3 mg/100 g, close to the value reported by (Islam et al., 2016). The extractability of iron in each beverage sample was determined as a percentage of this value.

**Fig. 1:**
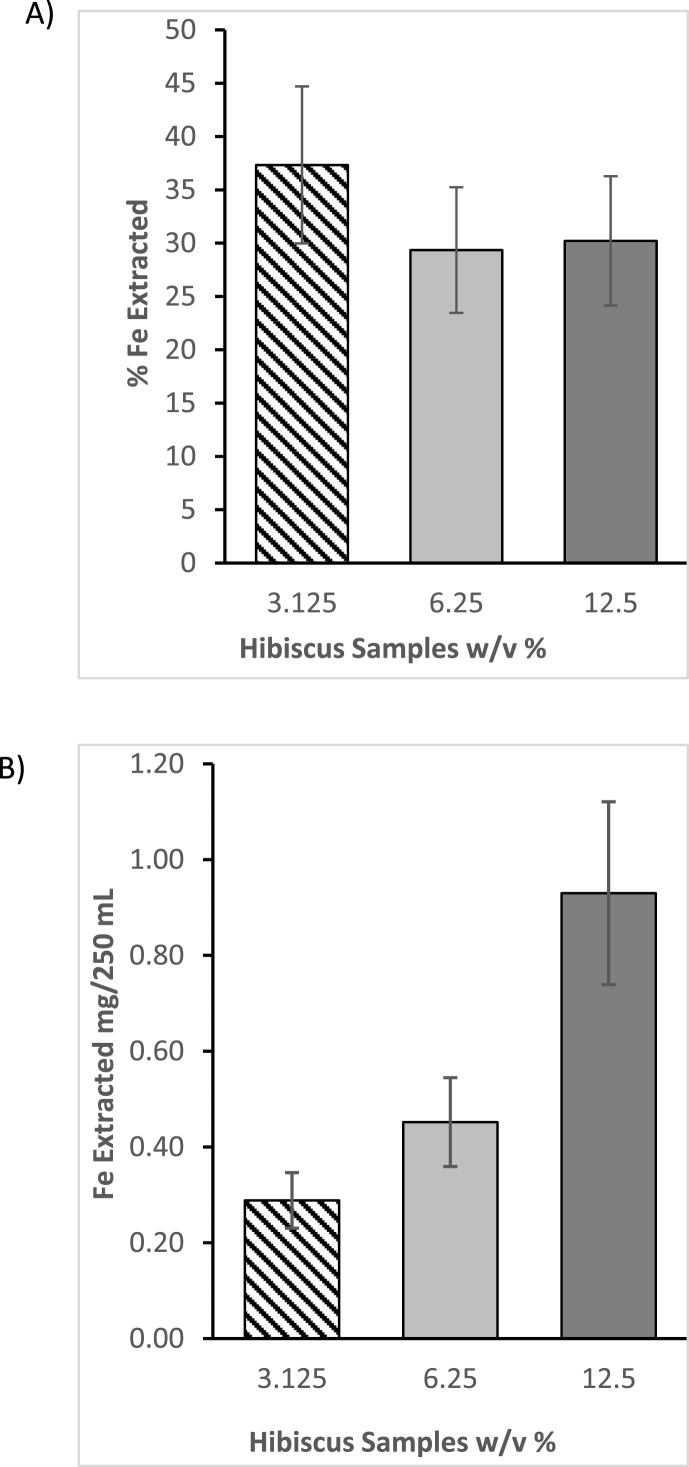
Iron extractability of 3.125, 6.25 & 12.5% w/v of hibiscus beverage. A) Expressed as the percentage of iron extracted; B) Expressed as total iron extracted into 250 mL of the beverage, Results are expressed as mean ± SD, n = 4.

Only 30–40% of the iron in the calyces was extracted (Fig. 1A), suggesting that most of the iron remained bound to the residual solids. Under the conditions tested, the average extractability of iron was 31.8 ± 6.7%, indicating that on its own, the hibiscus beverage is insufficient to curb iron deficiency significantly. This might also explain why no known studies validate the beverage's effectiveness in addressing ID among populations that cultivate *H. sabdariffa* L*.* and consume it in the form of a beverage, despite the relatively high iron content values reported for the plant calyces: 0.04–8.98 × 10^3^ mg/100 g (Abubakar et al., 2016).

Fig. 1B shows the estimated quantity of iron extracted into 250 mL, the assumed daily consumption volume of the beverage. Iron extractability remained approximately constant as the weight of the pulverized calyces increased.

### 3.2 Effects of different extraction conditions on iron and polyphenolic yield

**Fig. 2:**
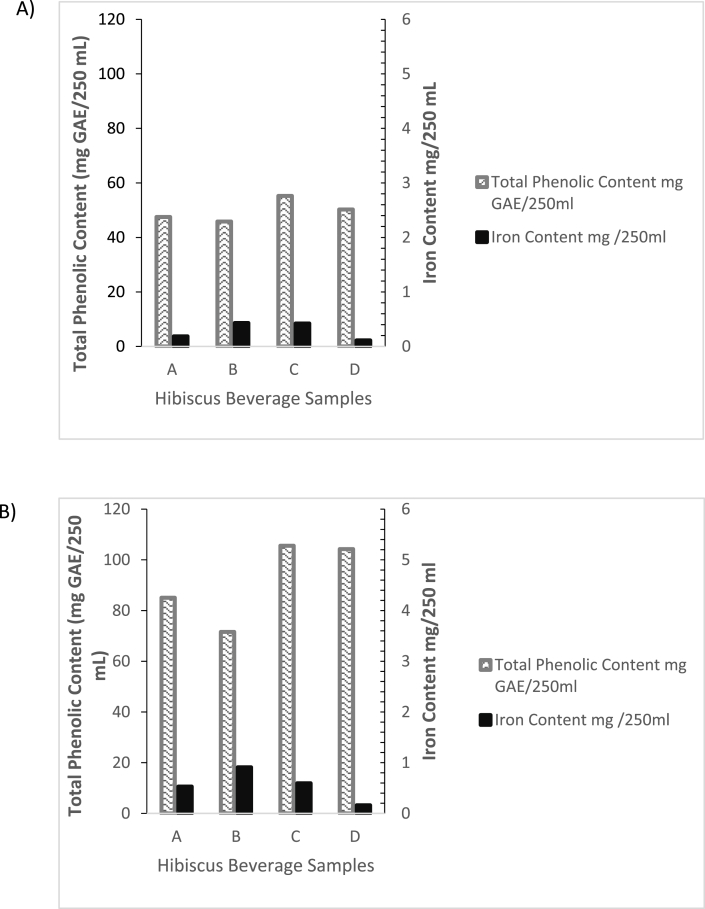
Iron and polyphenolic yield A) Lower-level concentration w/v: 6.25% B) Higher-level concentration w/v: 12.5% Extraction Conditions - A: 90 °C, steep time of 24 h; B: 90 °C, steep time of 0.5 h; C: RT, steep time of 24 h; D: RT, steep time of 0.5 h. For A and C heat treatment (boiling) was for 0.5 h.

Apparently, no published study has standardized hibiscus extraction to obtain the highest iron yield while concurrently maintaining the polyphenolic yield at a commercially acceptable level. The latter is pertinent because polyphenols impart desirable organoleptic properties like flavor, color, and astringency (De Oliveira et al., 2014, Lesschaeve and Noble, 2005) and confer therapeutic benefits as well (Sáyago-Ayerdi et al., 2007). Therefore, the effect of the concentration of the solid calyces (w/v%), temperature, and steep time on the extraction of iron and polyphenols were determined by a 2^3^ full factorial experiment. The results are summarized in Fig. 2A and 2B.

Maximum iron yields were achieved when the calyces were boiled in water for 0.5 h at an extraction temperature of 90 °C and then left to steep, off heat, for 0.5 h (condition B). The iron yields were 1.72 x 10^−3^ mg/mL and 3.64 x 10^−3^ mg/mL at the 6.25% (lower) and 12.5% (upper) concentration levels respectively. This supports a previous study's finding that heat treatments free up bound iron in plant-food sources (Hemalatha et al., 2007). However, condition B resulted in the lowest polyphenolic yields at both the upper and lower concentration levels, possibly the result of degradation due to the high extraction temperature.

Conversely, maximum polyphenolic yields were achieved when the extraction temperature was maintained at room temperature and the calyces were left steeping in water for 24 h (condition C). The recorded yields were 0.22 mg GAE/mL and 0.42 mg GAE/mL at the 6.25% and 12.5% concentration levels, respectively. This corroborated the sensory evaluation results reported by Ahmed et al. (2014), who found that lower extraction temperatures produced flavorful beverages that appealed more to their sensory panel participants. Under condition C, the iron yield observed at the lower concentration level, 6.25%, was comparable to the maximum iron yield at the same concentration level (observed under condition B). However, under the same condition C, for the higher concentration level of 12.5% the iron yield was about 35% lower than the maximum at the same concentration level (observed under condition B). Suggesting that heat treatment becomes a required factor for high iron yields during extraction as the beverage's w/v% concentration increases.

Comparing conditions A and B carried out at the higher temperature of 90 °C but at different steep times (off heat): 24 h and 0.5 h, respectively, it was observed, interestingly, that the iron yields were lower under condition A. Possibly, under condition A, as the mixture cooled down, over 24 h, some of the iron was again bound up in the compounds that had earlier dissociated due to the high temperature 90 °C.

The lowest iron yields, at both upper and lower concentration levels, were observed under condition D when the extraction temperature was maintained at room temperature in a water bath and left steeping for 0.5 h. However, the polyphenolic yields under this condition were comparable to those observed under condition C, suggesting that most of the flavor would be extracted by 0.5 h of steeping the hibiscus calyces without heat.

In summary, if obtaining optimum organoleptic properties were the focus of this study, then these results indicate that the beverage extraction should be carried out at room temperature for at least 0.5 h. However, for maximum iron extraction from the hibiscus calyces, which is one of the objectives of this study, 90 °C and a steep time of 0.5 h i.e., condition B, is preferred.

### 3.3 Selection of extraction conditions

Generally, maintaining the organoleptic properties of food products during processing is critical for high consumer acceptability (Darnton-Hill et al., 2017), and this should be kept in view also when improving food functionality. Accordingly, the polyphenolic contents of the samples with the highest iron yields, at 6.25% and 12.5% weight/volume, were compared to those of two commercially marketed beverages. Iron yields were also compared for benchmarking.

**Fig. 3:**
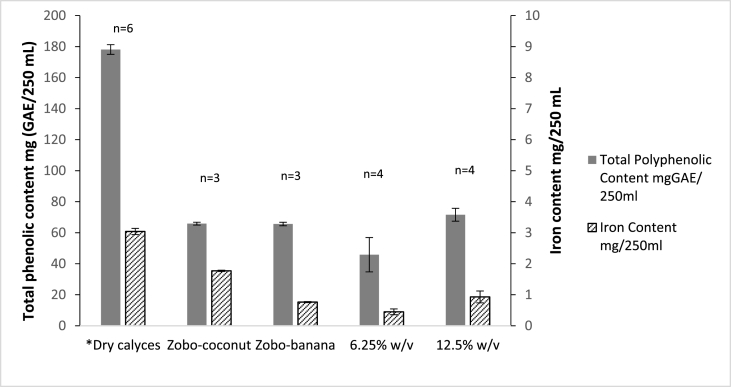
The polyphenol and iron content in *Dry calyces, 2 commercial hibiscus beverages and 2 lab-produced hibiscus beverage samples. Results expressed as mean ± SD. *Expected yields assuming all the iron and polyphenol content in the dry hibiscus calyces were transferred into a 12.5% w/v concentration beverage.

Fig. 3 shows that the concentration of polyphenols in the 12.5% beverage sample was ∼10% higher than the polyphenolic contents of both commercial hibiscus beverages, Zobo-coconut and Zobo-banana. Conversely, the polyphenolic content of the 6.25% beverage sample was ∼30% lower than the commercial ones. Therefore, the organoleptic properties of the 12.5% beverage are closer to those of the commercial beverages and might be more acceptable to the consumers.

Comparing iron contents in Fig. 3, the iron contents of the two commercial beverages were quite different, with one being lower and the other higher than the 12.5% hibiscus beverage sample. This might be due to the additional ingredients in the two commercial beverages or the natural variability of the hibiscus calyces composition.

Ultimately, the 12.5% extract obtained with 0.5 h steeping at 90 °C was selected for further study. Next, the iron and polyphenol yield from this sample were compared to the expected yield, assuming all the iron and polyphenolic content of the dry hibiscus calyces (*Dry calyces sample in Fig. 3) were transferred into a 12.5% w/v concentration beverage. Thus, the sample's iron extractability was determined to be ∼30% and polyphenol extractability ∼40%, i.e., 250 mL of the 12.5% sample contained 0.93 ± 0.19 mg iron and 71.58 ± 4.15 mg GAE polyphenols. The molar ratio of iron to polyphenols in the beverage was estimated to be 1:25. This molar overage indicates that the hibiscus beverage contains enough polyphenols to potentially bind both the native iron and iron added as a fortificant, forming the unabsorbable chelation complex. Therefore, the bioaccessibility of the iron in the fortified hibiscus beverage could be impacted.

### 3.4 Evaluation of iron-polyphenol complexes using spectrophotometry

McGee and Diosady's (2018b) method for quantifying the chelation complex formed between black tea polyphenols and added iron was adopted and adapted for this study. The method was used to quantify the complex formed by both the native iron and added iron in the *H. sabdariffa* L*.* calyces’ beverage. Reportedly, the effect of polyphenols on iron absorption depends on individual plant compositions and the quantity of polyphenols in the plant (Hurrell & Egli, 2010), therefore, it was expedient to evaluate the complexation in the hibiscus beverage.

It was assumed that iron-polyphenol chelation complex concentrations, represented by absorbance peaks, are directly related to the bioaccessibility of the iron in the hibiscus beverage samples. Absorbance readings were taken at 560 nm to match the peak wavelength of the absorbance scans of the standards used to generate the calibration curves for both sample sets: the unfortified and fortified hibiscus beverages.

**Fig. 4:**
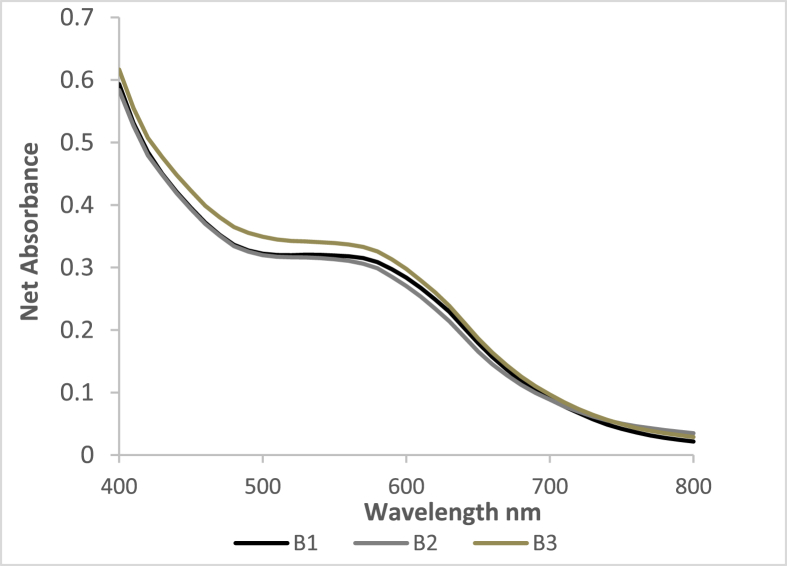
Spectrophotometric scans of 3 samples of hibiscus beverage (B1, B2 & B3) (pre-fortification) at pH 6.5.

**Fig. 5:**
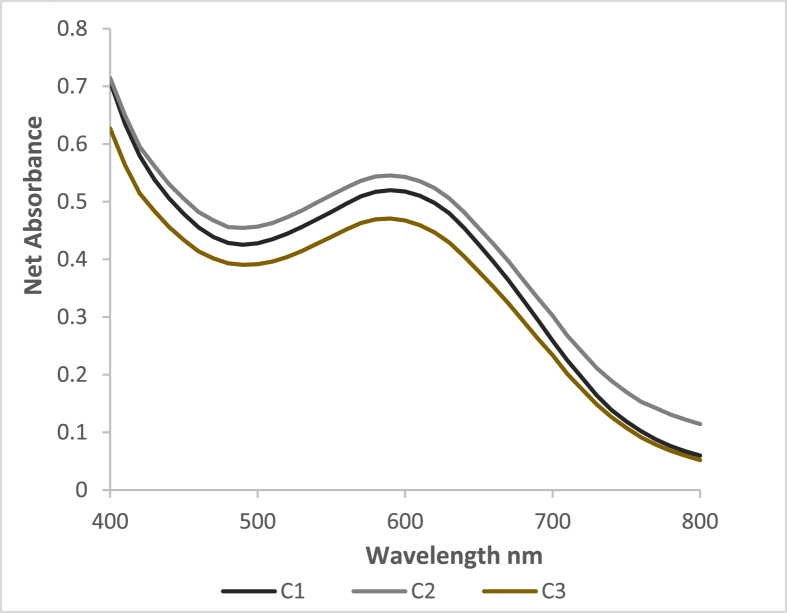
Spectrophotometric scans of 3 samples of fortified hibiscus beverage (C1, C2 & C3) at pH 6.5.

Fig. 4, Fig. 5 are the absorbance scans of 3 replicate samples of the hibiscus beverage pre-fortification and the fortified hibiscus beverage, respectively.

Fig. 4 shows a noticeable inflection at the expected wavelength range for iron-polyphenol complexes (Perron et al., 2010). Iron-polyphenol complexes are formed under less acidic to neutral conditions because, in the presence of iron cations the de-protonation of polyphenols is favored, leading to an oxygen center with a high charge density that promotes the chelation process (Hider et al., 2001). Therefore, it may be inferred that at the site of iron absorption, in the GI tract, where the pH range is between 6 and 7, the bioaccessibility of native iron in the beverage is inhibited by its polyphenols.

The scans of the fortified hibiscus beverage samples show more apparent peaks representing the iron-polyphenol complexes (Fig. 5). Furthermore, the shape of each of the three absorbance scans is similar to the absorbance scans reported for black tea iron-polyphenol complexes (McGee & Diosady, 2018a). Therefore, we may also infer that the bioaccessibility of the added iron in the beverage is inhibited by the polyphenols present in the beverage.

Table 2 presents the estimated quantities of the iron-polyphenol complexes formed in the beverage samples. Interestingly, the values recorded for the pre-fortification hibiscus beverage sample (sample B) are well above the expected maximum value of 0.07 mM FeSO_4_/GAE (estimated from the sample's pre-determined iron content). Suggesting that other unrelated interactions contributed to the higher absorbance value recorded within the wavelength range of interest. This is not unusual when analyzing biological materials, which are generally regarded as complex compounds. Besides, other compounds, apart from polyphenol complexes, also record absorbance readings in this wavelength region (Perron et al., 2010).

**Table 2:** Estimated quantity of iron-polyphenol complex formed at pH 6.5

Labels	Sample	Mean Absorbance at 560 nm^a^	Iron-polyphenol complex formed (mM FeSO_4_/GAE)	Iron content (mM)
C	Fortified beverage	0.49 ± 0.04	0.27 ± 0.02	0.43
B	Hibiscus beverage (pre-fortification)	0.32 ± 0.01	0.18 ± 0.01	0.07^b^
D = C - B	Added iron only	0.17 ± 0.05	0.09 ± 0.02	0.36^c^

B is the mean ± SD of B1, B2 & B3: replicates of the beverage samples pre-fortification; C is the mean ± SD of C1, C2 & C3: replicates of the fortified beverage samples; D it the mean of the differences between concurrent replicate samples.

a Wavelength of absorbance peaks used for calibration curve.

b Estimated from ICP OES.

c Ferrous sulfate added by direct mixing.

Iron, not bound in iron-polyphenol complexes, would probably exist in another complex form within the beverage samples. This can be attributed to its electronic configuration which gives iron an affinity for redox reactions and chelation. Iron complexes are known to be colored due to the empty d-orbitals typical of transition metals; thus, other iron complexes could interfere with the absorbance readings. Furthermore, given that like iron, other transition metals also have the propensity to form chelation complexes with polyphenols (Hider et al., 2001), particularly after the deprotonation of the polyphenols, the high absorbance values could also stem from other metal-polyphenol complexes. Notably, cultivation sites can influence the hibiscus calyces' chemical composition; therefore, depending on the vicinity's prevailing human activities and soil composition, other transition metals, including contaminants like mercury (Hg) and lead (Pb), could be found in the beverage.

**Fig. 6:**
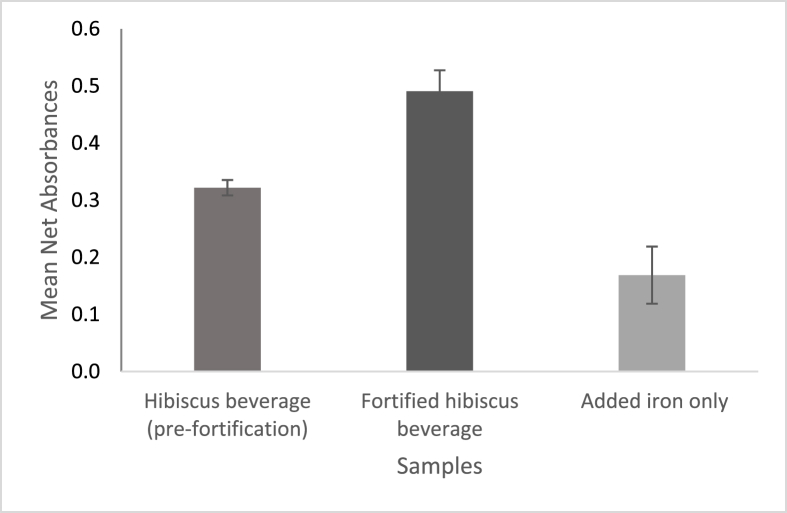
Mean net absorbance of the 2 beverage samples and their difference which represents the added iron only, at wavelength 560 nm. Results expressed as mean ± SD.

Fig. 6 demonstrates the higher-than-expected value estimated for the unfortified hibiscus beverage sample relative to the fortified beverage sample. The difference in the absorbance readings between the two beverage samples was determined to depict the discrepancy more clearly. This difference represents the estimated quantity of the chelation complex formed by polyphenols and the added iron only (sample D). The ratio of the molar concentration of the beverage's native iron content to that of the added iron is 1:5. However, the ratio of the estimated quantity of complex formed by the native iron relative to the added iron is 1: 0.5. Indicating that there are indeed other compounds/complexes contributing to the high absorbance reading and invariably, the high iron-polyphenol complex value reported for the hibiscus beverage (pre-fortification).

Nonetheless, the observed color change after the pH value of the samples were adjusted with NaOH, confirmed the presence of iron-polyphenol complexes. Therefore, with the discrepancy in the expected maximum value versus the estimated value, this model may not be suitable to quantitatively evaluate the iron-polyphenol complex formed in the hibiscus beverage pre-fortification. However, the model still allows the interaction to be evaluated qualitatively, as shown in Fig. 4.

Conversely, the model can be used to estimate the quantity of iron-polyphenol chelation complex formed between the beverage's native polyphenols and the added iron alone (sample D), the estimated quantity presented in Table 2 is 0.092 ± 0.022 mM FeSO_4_/GAE. This value suggests that not all the added iron in the fortified beverage was bound in the chelation complex. By comparing this estimated quantity to the expected maximum quantity after adding 0.358 mM of ferrous sulfate: 0.358 mM FeSO_4_/GAE, we can deduce that a substantial amount, ∼75%, remained unbound and is potentially bioaccessible.

The reported high ascorbic acid content in *H. sabdariffa* L. might have kept some of the iron in the fortified beverage unbound and, therefore, possibly available for absorption, even in the presence of polyphenols. Volkova et al. (2021) reported 18.4 mg of Vitamin C per 100 g of the dry plant part, and they suggested this contributes enormously to a synergism that limits the inhibitory effect of the plant's native polyphenols. Hurrell and Egli (2010) also reported that the presence of ascorbic acid can negate the inhibitory effect of polyphenols on both native and added iron. Additionally, it has been reported that the cancelling effect for non-heme iron depends on the quantity of ascorbic acid relative to the polyphenolic content (Siegenberg et al., 1991). Invariably, the possibility that some of the iron in the fortified hibiscus beverage might be available for absorption during digestion is significant. This has implications for the suitability of the fortified beverage as an iron delivery vehicle and its potential to contribute to the prevention/reduction of ID among vulnerable populations.

### 3.5 Study relevance and its implication for the prevalence of ID in sub-Saharan Africa

Addressing iron deficiency at the population level in sub-Saharan Africa has proven particularly challenging. A reason Mwangi et al. (2021) provided for this in their review paper was the substantial presence of iron inhibitors in the region's staple foods, leading to low bioaccessibility of iron in their diets. One such staple is the hibiscus beverage, and evaluating its potential as a valuable source of iron could serve as a baseline study for assessing the viability of other staples, particularly those rich in polyphenols.

This study's findings would help fill an identified gap in the strategies currently available to address the prevalence of ID in the SSA region: the need for more sustainable iron fortification initiatives adapted to the local context and consumption patterns of the most at-risk demographics. The expected outcome is the use of results from this study and similar studies as springboards for future studies on solutions that make polyphenol-rich staples efficacious iron delivery systems.

## 4 Conclusion

From this study, it can be deduced that a low iron extractability of 31.8 ± 6.7%, which resulted in ∼1 mg Fe/250 mL of the hibiscus beverage, renders the unfortified beverage incapable of significantly impacting the prevalence of iron deficiency among consumers. Especially as iron-polyphenol complexes, shown to be formed during the digestion process, only made the iron content of the unfortified beverage less bioacessible. Similarly, the results showed that the bioaccessibility of the iron, added as a fortificant to the beverage to attain the targeted concentration of 6 mg per 250 mL, is also impacted by the formation of iron-polyphenol complexes. However, the estimated quantity of iron-polyphenol chelation complex formed, 0.092 ± 0.022 mM FeSO_4_/GAE, is only a quarter of the amount that could have been formed had all the added iron, 0.358 mM of ferrous sulfate, been bound in the chelation complex. Therefore, ∼75% of the added iron remained unbound and thus was available for absorption. This indicates that, if more iron is added, the fortified hibiscus beverage could deliver the targeted quantity of iron in a bioaccessible form.

Nonetheless, since most of the SSA populace is predominantly dependent on plant-based diets, which are rich in iron inhibitors, it is imperative to improve the bioaccessibility of the iron content of the fortified hibiscus beverage. Especially as iron inhibitors present in food matrices consumed along with it could impact the bioaccessibility of the beverage's iron content, thus reducing the fortified hibiscus beverage's effectiveness as an iron source.

Strategies to improve the bioaccessibility of the iron content of the fortified hibiscus beverage include introducing ascorbic acid that prevents iron-polyphenol complexation. Also, an alternative iron source, such as a chelated iron source with proven bioaccessibility in the presence of iron inhibitors, e.g., ferric sodium EDTA, could be considered in place of ferrous sulfate. However, the cost of these strategies must not impact on the ability of the most vulnerable to afford the fortified hibiscus beverage. Ultimately, the value of the unfortified and fortified hibiscus beverages as iron sources could be significantly improved if the complexing of iron by native polyphenols could be reduced or prevented.

## CRediT authorship contribution statement

**Ade O. Oyewole:** was responsible for the conceptualization, methodology, experimentation, and the writing of this manuscript.

**Levente L. Diosady:** supervised the experimentation, he also reviewed and edited the manuscript.

Both authors have approved the submission of this manuscript.

## Funding sources

This research was supported by the 10.13039/100002322Schlumberger Foundation via their flagship program: the Faculty for the Future Fellowship and the 10.13039/100000865Bill & Melinda Gates Foundation (OPP1151531).

## Declaration of competing interest

The authors declare that they have no known competing financial interests or personal relationships that could have appeared to influence the work reported in this paper.

## Data Availability

Data will be made available on request.
